# Supplementary material for: Mechanisms governing the pioneering and redistribution capabilities of the non-classical pioneer PU.1
Source: Nat Commun. 2020 Jan 21;11:402. doi: 10.1038/s41467-019-13960-2 (PMC6972792; doi:10.1038/s41467-019-13960-2)
Supplement: Supplementary file 7 — Source data [file 41467_2019_13960_MOESM7_ESM.zip › Source_Data/Figure6/Figure6B_MotifScanOutput/FLI1_mutPU.1/homerResults/motif43.similar.html]

motif43

## Information for motif43

T
G
C
A
T
A
G
C
G
A
C
T
G
T
C
A
G
T
A
C
T
C
G
A
T
G
C
A
G
A
C
T
G
C
A
T
G
A
T
C
T
G
A
C
G
A
T
C
  
Reverse Opposite:  

C
T
A
G
A
C
T
G
C
T
A
G
C
G
T
A
C
T
G
A
A
C
G
T
A
G
C
T
C
A
T
G
C
A
G
T
C
T
G
A
A
T
C
G
A
C
G
T
  

|  |  |
| --- | --- |
| p-value: | 1e-25 |
| log p-value: | -5.805e+01 |
| Information Content per bp: | 1.710 |
| Number of Target Sequences with motif | 162.0 |
| Percentage of Target Sequences with motif | 1.58% |
| Number of Background Sequences with motif | 187.1 |
| Percentage of Background Sequences with motif | 0.49% |
| Average Position of motif in Targets | 109.2 +/- 58.3bp |
| Average Position of motif in Background | 94.0 +/- 63.3bp |
| Strand Bias (log2 ratio + to - strand density) | -0.3 |
| Multiplicity (# of sites on avg that occur together) | 1.16 |
| Motif File: | file (matrix) reverse opposite |

### Similar de novo motifs found

|  |  |  |  |  |  |  |  |
| --- | --- | --- | --- | --- | --- | --- | --- |
| Rank | Match Score | Redundant Motif | P-value | log P-value | % of Targets | % of Background | Motif file |
| 1 | 0.934 | G A T C A G C T A C T G C T A G A T C G C G T A C T G A A G C T G A C T A C T G A C G T C T G A A T C G A C G T | 1e-19 | -44.618260 | 0.82% | 0.18% | motif file (matrix) |
| 2 | 0.931 | T C G A T G C A A G T C C G A T T G C A A T G C G T C A C T G A A G T C A G C T G T A C G A T C G A T C | 1e-18 | -43.107079 | 0.96% | 0.26% | motif file (matrix) |
